# Supplementary material for: An ultrasensitive and broadband transparent ultrasound transducer for ultrasound and photoacoustic imaging in-vivo
Source: Nat Commun. 2024 Feb 16;15:1444. doi: 10.1038/s41467-024-45273-4 (PMC10873420; doi:10.1038/s41467-024-45273-4)
Supplement: Supplementary file 1 — Supplementary Information [file 41467_2024_45273_MOESM1_ESM.pdf]

Supplementary Information for "An ultrasensitive and broadband transparent ultrasound transducer for ultrasound and photoacoustic imaging in-vivo"

Seonghee Cho[1,2†], Minsu Kim[2,3†], Joongho Ahn[2,3], Yeonggeun Kim[2,3], Junha Lim[4], Jeongwoo Park[2,3], Hyung Ham Kim[1,2,3], Won Jong Kim[4,5], Chulhong Kim[1,2,3,5,6]*

[1] Department of Electrical Engineering, Pohang University of Science and Technology, Pohang, Republic of Korea.

[2] Medical Device Innovation Center, Pohang University of Science and Technology, Pohang, Republic of Korea.

[3] Department of Convergence IT Engineering, Pohang University of Science and Technology, Pohang, Republic of Korea.

[4] Department of Chemistry, Pohang University of Science and Technology, Pohang, Republic of Korea.

[5] Department of Medical Science and Engineering, Pohang University of Science and Technology, Pohang, Republic of Korea.

[6] Department of Mechanical Engineering, Pohang University of Science and Technology, Pohang, Republic of Korea.

*chulhong@postech.edu

† These authors contributed equally to this work.

Contents

List of abbreviations.
Supplementary Figure 1. Gain response and pressure transmittance on the frontside of a piezo crystal
Supplementary Note 1. Effect of front layer combination on transfer pressure gain and transducer performance
Supplementary Note 2. Recommended acoustic impedance of the backing layer for optimum bandwidth
Supplementary Figure 2. Scheme for finding an appropriate matching material design
Supplementary Note 3. Common configuration of a conventional opaque ultrasound transducer
Supplementary Figure 3. Comparisons of the effective front loads and phase angles in previously reported studies.
Supplementary Note 4. Comparisons of simulated acoustic impedance properties of reported transparent ultrasound transducers (TUTs) and a proposed TUT
Supplementary Table 1. Elastic/shear moduli and densities of the epoxy matrix and ceramic filler particles used in the acoustic velocity and impedance simulations
Supplementary Table 2. Acoustic parameters for all components used in the proposed TUT
Supplementary Figure 4. Comparison of the simulated electric input impedances of the proposed transparent ultrasound transducer (TUT), a conventional opaque ultrasound transducer (OUT), and a conventional TUT.
Supplementary Table 3. The theoretical lateral and axial resolution of the ultrasound (US)/photoacoustic (PA) imaging system and its measured resolution.
Supplementary Figure 5. Simulated horizontal slice of point spread function (PSF) generated by the square aperture for ultrasound (US) and photoacoustic (PA) imaging and lateral and diagonal line profiles of PSF
Supplementary Figure 6. B-scan of ultrasound (US) and photoacoustic (PA) image for signal-to-noise ratio measurement
Supplementary Note 5. Detailed depth-wise analyses of ultrasound (US) and photoacoustic (PA) projection images of a mouse
Supplementary Note 6. Detailed depth-wise analyses of ultrasound (US) and photoacoustic (PA) cross-section images of a mouse.
Supplementary Figure 7. Measured efficiency $\eta(f)$ spectra of various ultrasound (US) transducers.
Supplementary Table 4. Performance benchmarks of various ultrasound (US) transducers.
Supplementary Table 5. Simulation parameters used to compare the point spread functions of the custom-made RUT and the proposed TUT.
Supplementary Figure. 8. Computational comparison of the acoustic point spread function (PSF) of the proposed transparent ultrasound transducer (TUT) and the ring-shaped ultrasound transducer (RUT).
Supplementary Figure. 9. Photoacoustic (PA) imaging of a character logo target phantom and a leaf skeleton target using the proposed transparent ultrasound transducer (TUT) and the ring-shaped ultrasound transducer (RUT)
Supplementary Figure. 10. Computational pulsed acoustic field comparison for an N-SF11 concave lens-focused transparent ultrasound transducer (TUT) and the proposed TUT with parabolic mirror focusing
Supplementary Figure. 11. Axial resolutions vs penetration depths of *in-vivo* photoacoustic microscopy studies.
Supplementary Table 6. Summary of performances of acoustic resolution photoacoustic microscopy in chicken breast tissue, a mouse, a rat, and a human palm.
Supplementary Note 7. Importance of acoustic numerical aperture in determining sensitivity and imaging depth
Supplementary references

List of abbreviations

Abbreviation	Paraphrase
AR-PAM	Acoustic-resolution photoacoustic microscopy
B-mode	Brightness mode
CNR	Contrast-to-noise ratio
DRR	Depth-to-resolution-ratio
LNO	Lithium niobite
OUT	Opaque ultrasound transducer
PA	Photoacoustic
PAI	Photoacoustic imaging
PAM	Photoacoustic microscopy
Q factor	Quality factor
RUT	Ring-shaped ultrasound transducer
SNR	Signal-to-noise ratio
TUT	Transparent ultrasound transducer
US	Ultrasound
USI	Ultrasound imaging

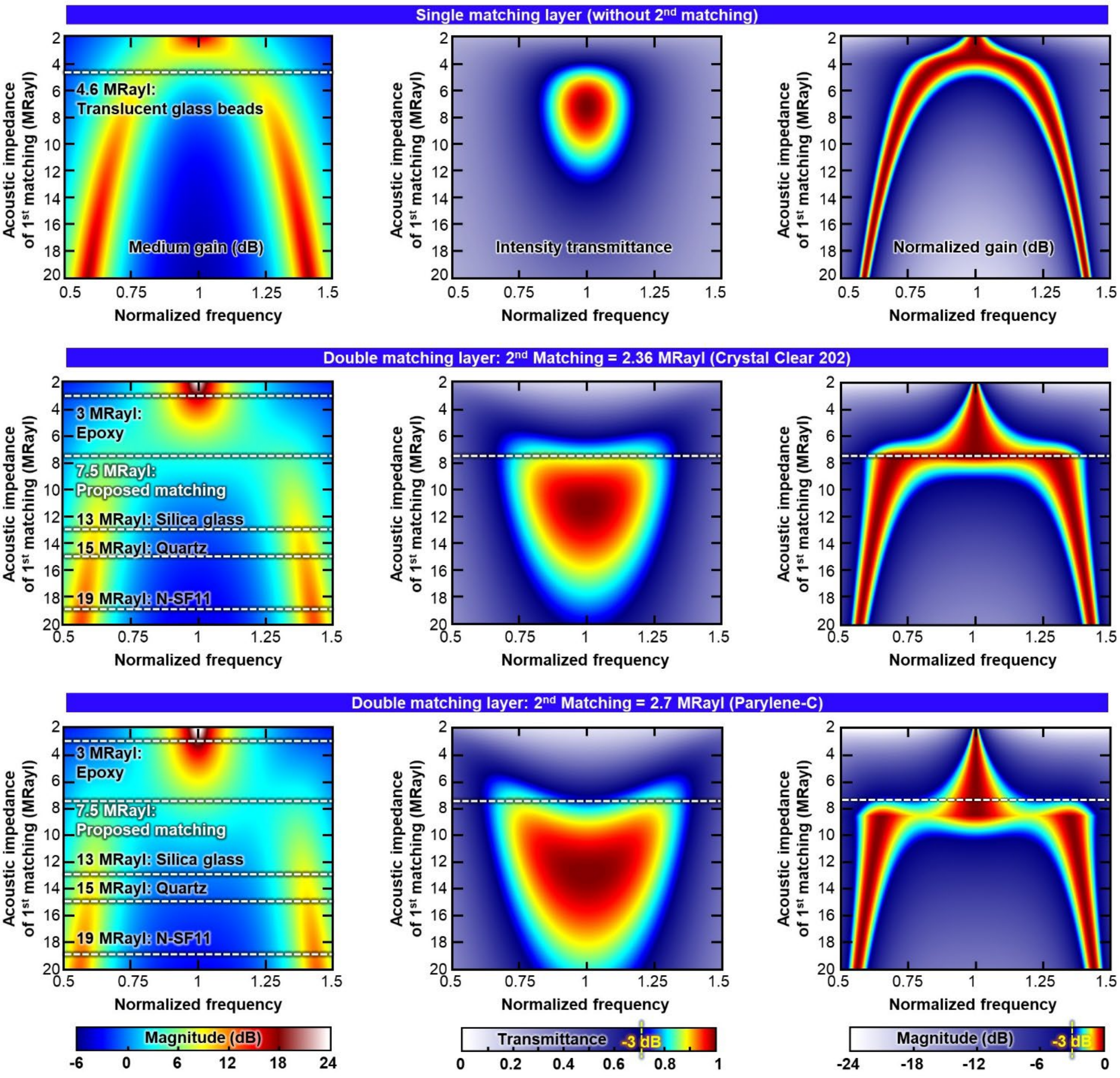

Supplementary Fig. 1. Gain response and pressure transmittance on the frontside of a piezo crystal

Supplementary Note 1. Effect of front layer combination on transfer pressure gain and transducer performance

Optimizing pressure amplification gain and pressure transmittance is crucial to enhancing the performance of contemporary TUTs. These factors depend on the difference in acoustic impedance between the gain medium and the transmission medium. A larger mismatch leads to higher pressure amplification but lower transfer efficiency, while a closer match results in lower pressure amplification but higher transfer efficiency. The critical challenge in TUT production is finding the right balance to achieve a transmit pressure gain that is both flat and high. These factors can be calculated by examining the reflectance of the front half of the acoustic transmission line from the centre of the piezo crystal. The pressure transmittance to the loading medium is determined by the following equation:

$$T_F = \sqrt{\frac{Z_F}{Z_C}(1-|\Gamma_F|^2)}, \qquad \Gamma_F = \frac{Z_{F_{in}} - Z_C}{Z_{F_{in}} + Z_C}, \tag{1}$$

where T_F is pressure transmittance from the piezo crystal to the front load medium, Z_F is the acoustic impedance of the front load medium, Z_{Fin} is the acoustic input impedance of the front half of the transducer, Z_C is the acoustic impedance of the piezo crystal, and Γ_F is the pressure reflectance caused by the front part of the transducer. Assuming that the front half of the transducer is connected to a perfectly reflective boundary, the resulting front pressure amplification gain (G_F) and transmitted pressure gain (G_{FT}) can each be expressed as the infinite sum of an infinite geometric series:

$$G_F = \frac{1}{1-\Gamma_F}, \qquad G_{FT} = G_F T_F. \tag{2}$$

The calculated results for several representative cases are depicted in Supplementary Fig. 1. As depicted, we have presented the normalized frequency response of gain and transmittance concerning the acoustic impedance variation of the 1st matching layer. In the left column, we show pressure amplification gain, pressure transmittance, and normalized transmitted gain. We conducted normalization based on the maximum and minimum value of the transmitted pressure gain for each acoustic impedance. Among the three illustrated cases, the 2.36 MRayl 2nd layer in the double matching structure exhibits the most

consistent response in normalized transmitted gain. In this instance, when the 1st layer has an impedance below 7 MRayl, the transmittance is low, and when the 2nd layer exceeds 9 MRayl, the amplification gain significantly diminishes. These outcomes appear due to narrow bandwidth and dual-frequency characteristics, respectively. Therefore, it is reasonable for the 1st layer to fall within the range of 7–9 MRayl, and we opted for 7.5 MRayl, which shows a highly flat response.

Supplementary Note 2. Recommended acoustic impedance of the backing layer for optimum bandwidth

According to Desilet et al.'s[1] analysis using the Krimholtz, Leedom, and Matthaei (KLM) model, the transducer has an optimum bandwidth when the acoustic Q factor (Q_a) and electric Q factor (Q_e) are balanced, and each Q factor can be estimated as follows.

$$Q_a = \frac{\pi Z_C}{2(Z_B + Z_F)}, \qquad Q_e = \frac{\pi(Z_B + Z_F)}{4k_t^2 Z_C}. \tag{3}$$

Here, k_t is the electromechanical coupling coefficient, and Z_B, Z_F, and Z_C represent the effective acoustic impedances of the backload, frontload, and gain medium. When water (1.5 MRayl) is the final load, the front load of our proposed TUT is 16.4 MRayl. For LNO, k_t is 0.49 and Z_C is 34.1 MRayl. In this case, the backload that equalizes Q_a and Q_e is 7.2 MRayl.

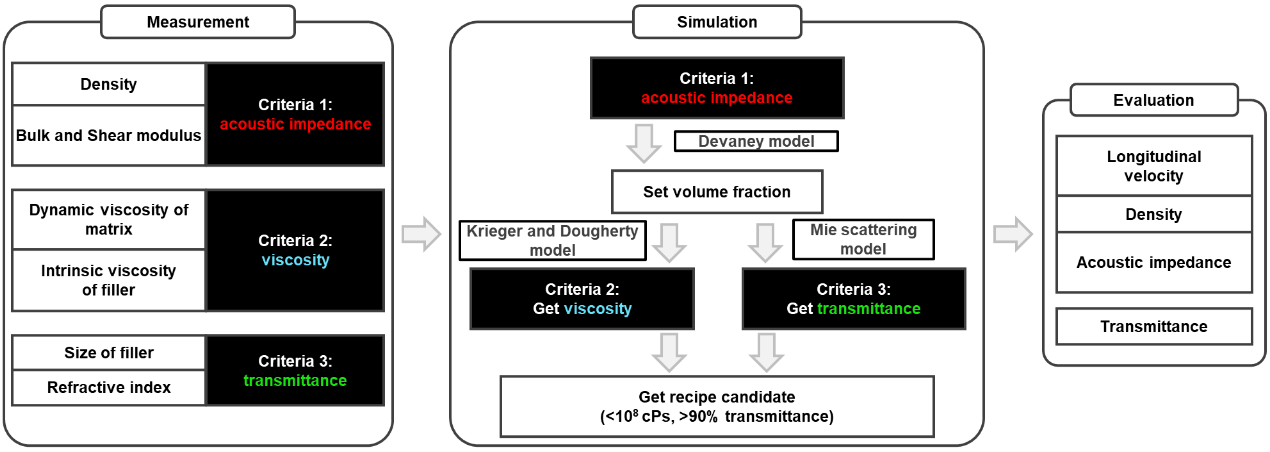

Supplementary Fig. 2. Scheme for finding an appropriate matching material design

Supplementary Note 3. Common configuration of a conventional opaque ultrasound transducer

As shown in Fig. 1a, a conventional opaque ultrasound transducer (OUT) comprises an opaque front quarter-wave double matching layer, a metal electrode, and a backing layer, with a piezo crystal in the middle. Each matching layer is a quarter of the center wavelength thick, whereas the piezo crystal is half a wavelength thick. The piezo crystal can be either transparent or not. In a conventional OUT, a metal-epoxy composite is widely used for the 1st front matching layer because its acoustic impedance is typically formulated to be 7–9 MRayl, and the easily mixed composite makes it simple to control the layer thickness and to connect wires[2]. On the backing side, a similar metal-epoxy composite is recommended, but with a more highly viscoelastic polymer matrix than in the front side layer, to efficiently absorb acoustic power[3,4]. The acoustic impedance of the backing side is set to be about 5–6 MRayl for adequate acoustic damping.

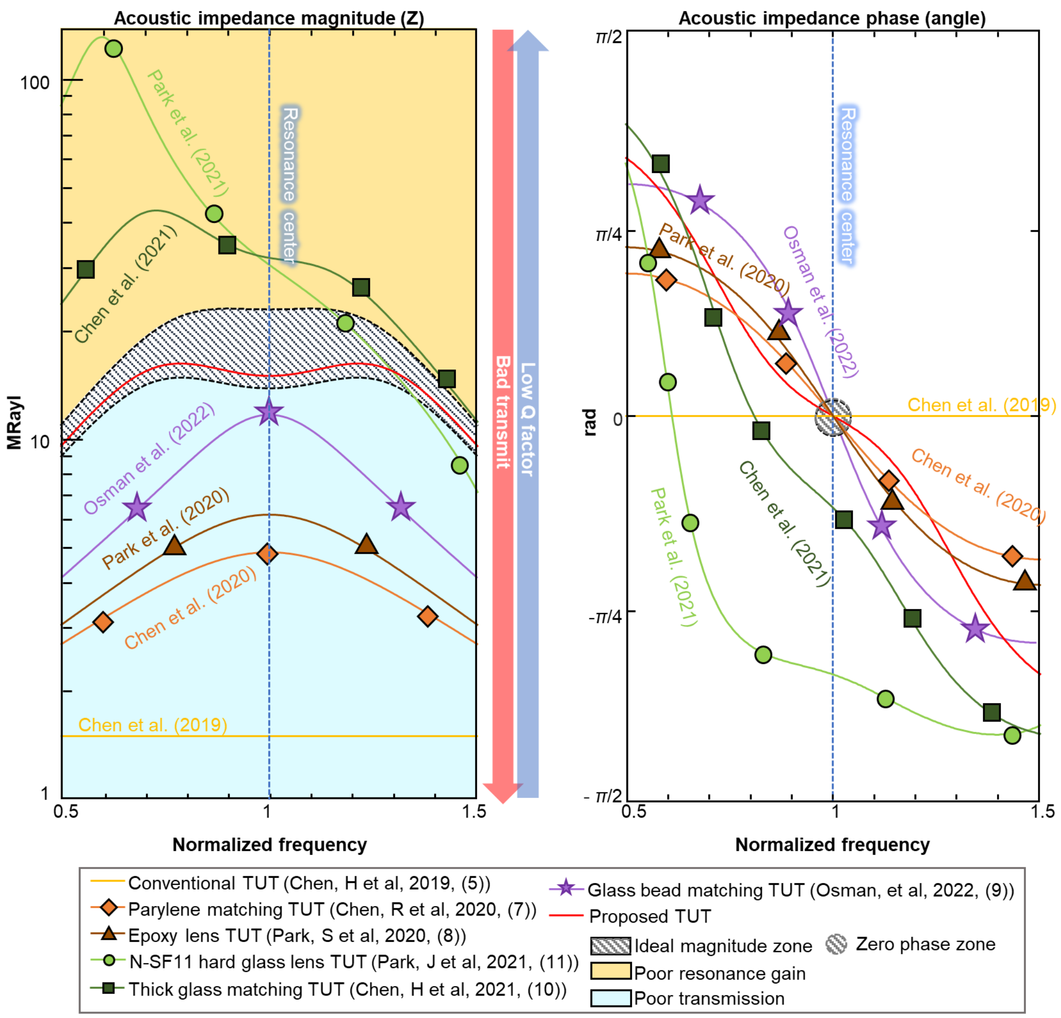

Supplementary Fig. 3. Comparisons of the effective front loads and phase angles in previously reported studies.

Supplementary Note 4. Comparisons of simulated acoustic impedance properties of reported transparent ultrasound transducers (TUTs) and the proposed TUT

Similar to the performance of a basic conventional transparent ultrasound transducer (TUT)[5,6] (the solid yellow line in Fig. 1b), TUTs with a single polymer matching layer (indicated by the solid orange line with diamond markers in Supplementary Fig. 3[7]) or with an epoxy lens (the solid brown line with triangle markers in Supplementary Fig. 3 [8]) suffer from poor acoustic transmission. While a TUT with a glass-bead-composite matching layer (the solid purple line with star markers in Supplementary Fig. 3[9]) exhibits good acoustic transmission compared to the other TUTs, its front-loading impedance is less than optimal. TUTs with a thick glass plate (the solid dark green line with squares in Supplementary Fig. 3[10]) or an N-SF11 glass lens (the solid light green line with circles in Supplementary Fig. 3[11]) have an unusually high input impedance as well as phase angle deviation at the zero-phase point, resulting in a low Q factor and consequent spectral mismatch.

Supplementary Table 1. Elastic/shear moduli and densities of the epoxy matrix and ceramic filler particles used in the acoustic velocity and impedance simulations

		Mechanical properties					Estimated results			
		Particle size [a]	Density (kg/m^3)	Bulk modulus (GPa)	Shear modulus (GPa)	Refractive index at 589 nm	Intrinsic viscosity	Max volume fraction (%)	Max acoustic impedance (MRayl)	Max usable impedance [b] (MRayl)
Matrix	EPO-TEK 301	-	1150	5.76	1.74	1.52	-	-	-	-
Ceramic filler	SiO_2 micro	3 µm	2650	37.80	44.40	1.54	8.68	67.27	8.43	7.46
	SiO_2 nano	15 nm	2200	36.68	31.10	1.46	28.97	21.92	3.96	3.81
	Al_2O_3 micro	5 µm	3974	251.92	161.61	1.77	4.53	67.39	17.46	16.95
	Al_2O_3 nano	25 nm	3974	251.92	161.61	1.77	26.99	27.49	5.33	4.76

[a] Median or average size, given by supplier.
[b] 10^8cPs is the maximum value for a usable composite

Supplementary Table 2. Acoustic parameters for all components used in the proposed TUT

Material	Longitudinal velocity (m/s)	Acoustic impedance (MRayl)
Lithium niobate (dielectric constant, 28; coupling coefficient, 0.49)	7340	34.13
Backside matching composite (33 wt % 3μm SiO_2)	2744	3.88
Frontside matching composite (77 wt % 3μm SiO_2)	3673	7.48
Crystal Clear 202	2280	2.36
Insulcast 502/ 2-3 μm silver composite	1900	7.33
E-Solder 3022	1850	5.92
EPO-TEK 301	2650	3.05
Parylene C	2141	2.76

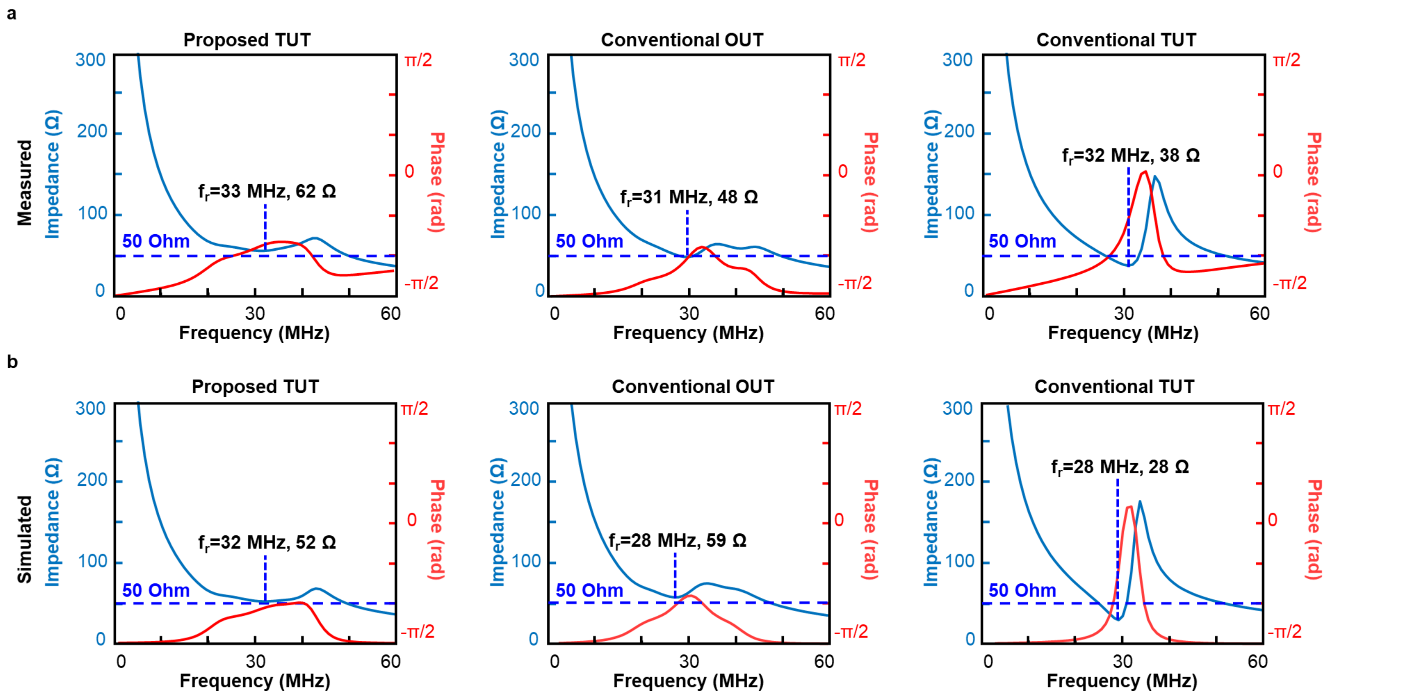

Supplementary Fig. 4. Comparison of the simulated electric input impedances of the proposed transparent ultrasound transducer (TUT), a conventional opaque ultrasound transducer (OUT), and a conventional TUT. a, Measured electrical impedance. **b,** Simulated electrical impedance.

Supplementary Table 3. Theoretical lateral and axial resolutions of ultrasound (US)/photoacoustic (PA) imaging system and measured resolution.

	Ultrasound microscopy	Acoustic resolution photoacoustic microscopy
Measured resonance frequency (f_A)	33 MHz	
Measured bandwidth (Δf_A)	19.59 MHz	27.46 MHz
Theoretical axial resolution	$\frac{0.88v_A}{2\Delta f_A} \approx 33\ \mu m$	$\frac{0.88v_A}{\Delta f_A} \approx 47\ \mu m$
Measured axial resolution	32.6 ± 1.9 μm	40.4 ± 1.6 μm
Lateral resolution estimated by acoustic pressure field simulation	102 μm – 145 μm	142 μm – 200 μm
Measured lateral resolution	110.4 ± 21.8 μm	148.4 ± 13.5 μm

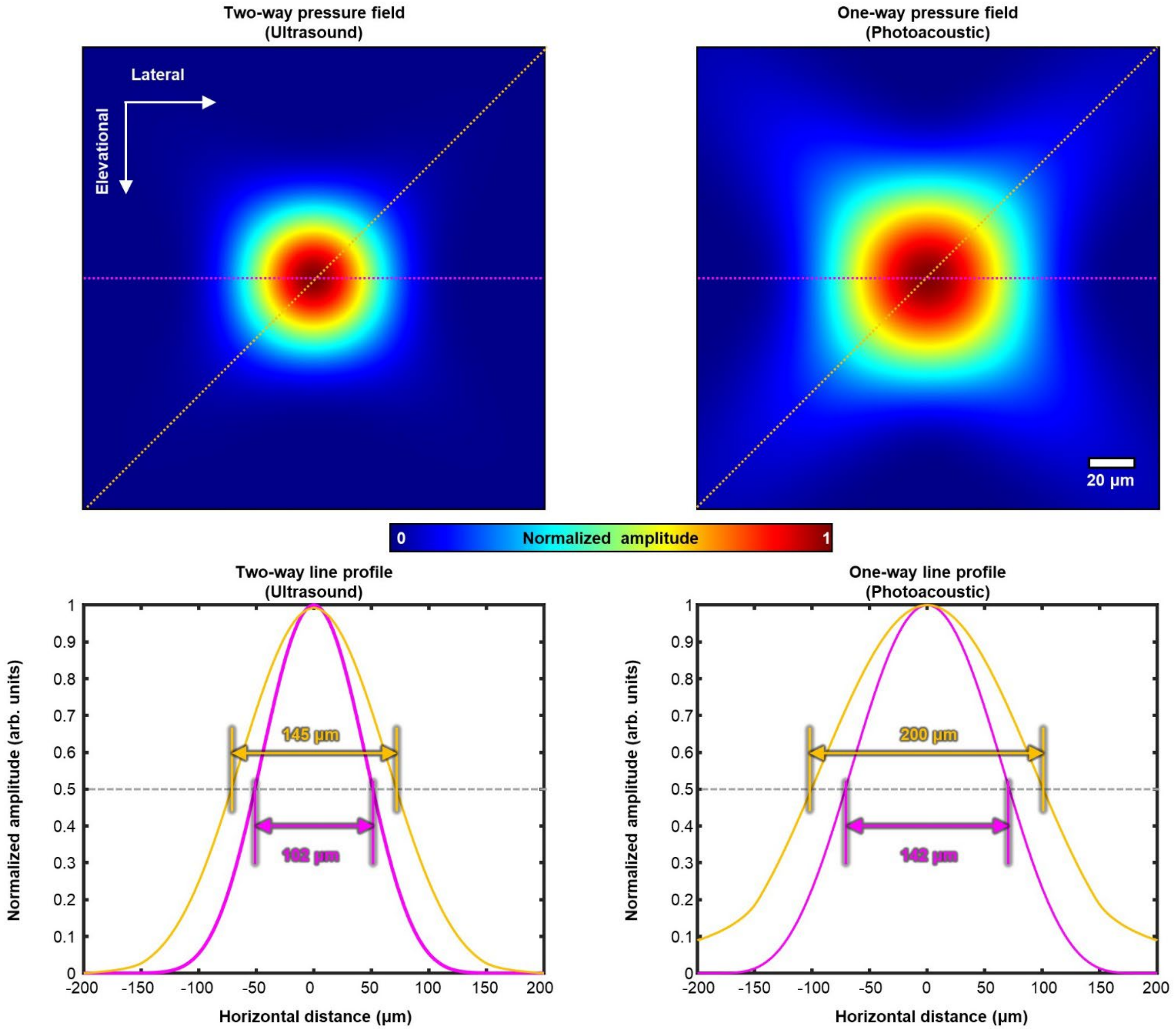

Supplementary Fig. 5. Simulated horizontal slice of point spread function (PSF) generated by the square aperture for ultrasound (US) and photoacoustic (PA) imaging and lateral and diagonal line profiles of PSF

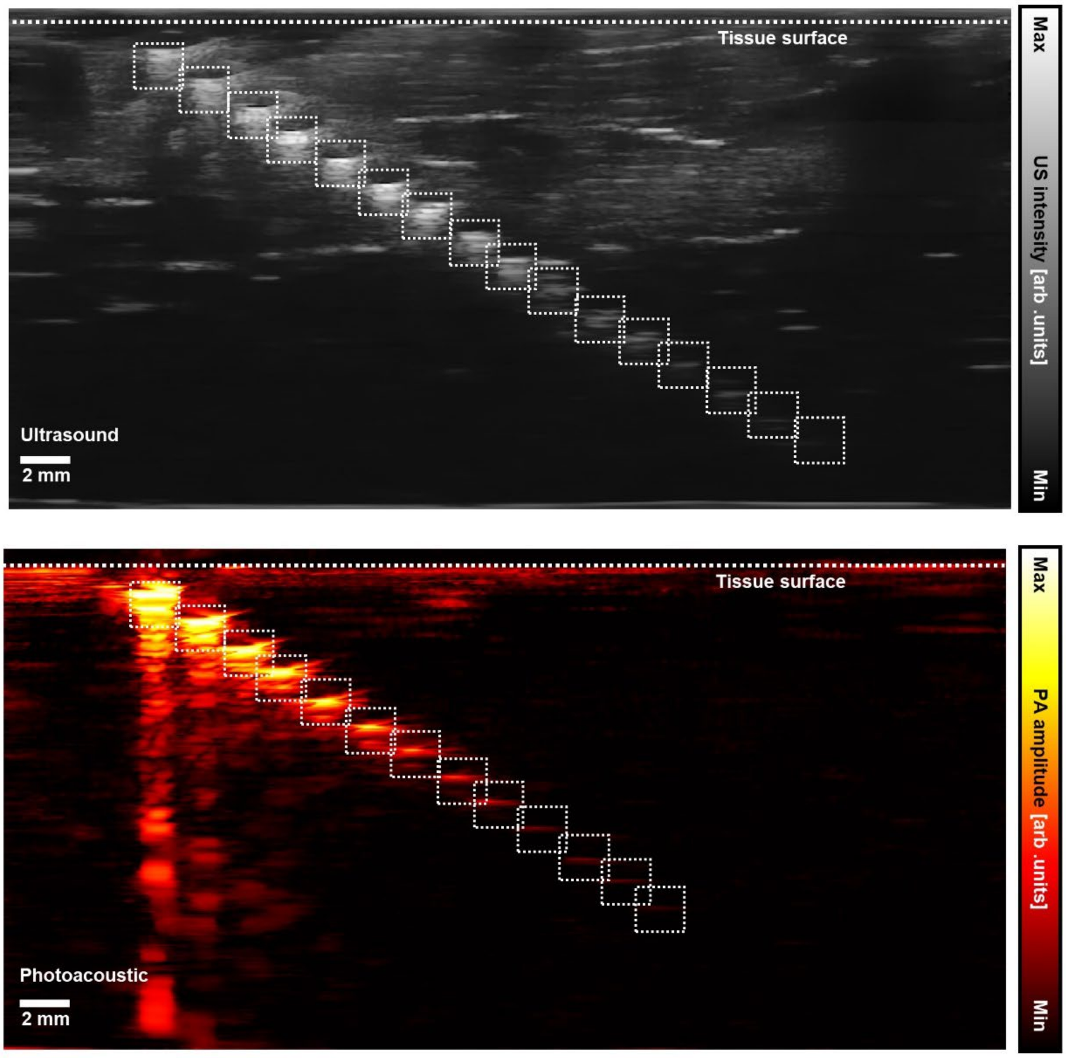

Supplementary Fig. 6. B-scans of ultrasound (US) and photoacoustic (PA) image for signal-to-noise ratio measurement White boxes indicate where targets (pencil lead with a 0.5 mm diameter) are located in the image.

Supplementary Note 5. Detailed depth-wise analyses of ultrasound (US) and photoacoustic (PA) projection images of a mouse.

In Fig. 5b, the depth-sliced thorax region ventral ultrasound (US) and photoacoustic (PA) images show details of the following murine blood vessels and organs: (1) the skin, sternum, and mammary/epigastric vessels at 0–1 mm, (2) the liver and major large blood vessels (e.g., common carotid, subclavian, and internal thoracic) at 0–2 mm, and (3) the heart and the bottom of the thorax region, including vertebra, at 2–11 mm and 11–15 mm, respectively. Superficial blood vessels and the gastrointestinal tracts, vertebra,

and the pelvis are more detailed in the abdominal region ventral depth-sliced US and PA images, which show (1) the mammary/epigastric vessel and superficial vessels on the intestinal and lower limb surfaces at 0–1 mm, (2) the gastrointestinal tract, preputial gland, and bladder at 1–11 mm, and (3) the bottom of the abdominal region, including the vertebra and pelvis, at 11–15 mm. The sensitive US and PA sagittal images describe the details of blood vessels, organs, and bones as follows: (1) the aterial thoracic and mammary/epigastric vessels at 0–1 mm, (2) the stomach, spleen, and kidney at 0–2 mm, and (3) the liver, cecum, vertebra, scapula, pelvis, and femur at 1–11 mm.

Supplementary Note 6. Detailed depth-wise analyses of ultrasound (US) and photoacoustic (PA) cross-section images of a mouse.

In Fig.6, the B-mode US image from L1 reveals the mid-thorax, including the thoracic cavity and surrounding structures such as the skin, sternum, and heart. The sinuses, sternum, vessels, and heart are visualized in the B-mode PA image. The B-mode US image at L2 shows the upper abdomen, including the spine, gallbladder, and stomach, while the PA image shows the top of these structures. At L3, the middle section of the abdomen (e.g., intestines) is displayed in the US image, while the abdominal vessels are in the PA image. At L4, the liver and surrounding ribs are delineated in the US image. At L5, the kidney and surrounding tissues are shown, whereas the spleen is prominent in the PA image.

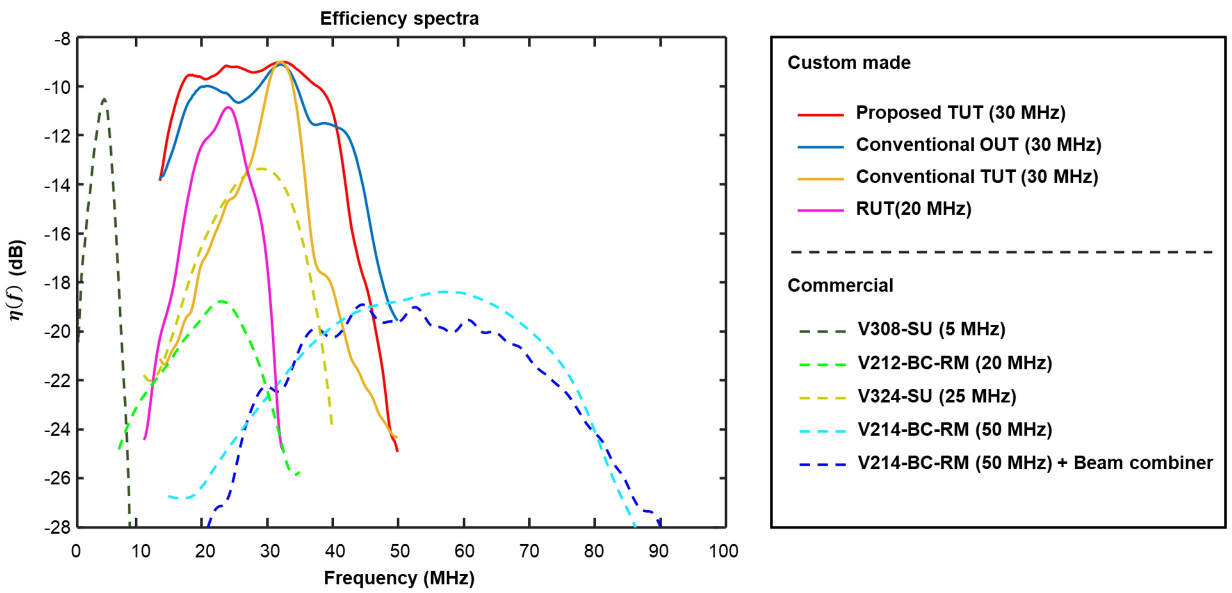

Supplementary Fig. 7. Measured efficiency *η(f)* spectra of various ultrasound (US) transducers. TUT, transparent ultrasound transducer; OUT, opaque ultrasound transducer; and RUT, ring-shaped ultrasound transducer.

Supplementary Table 4. Performance benchmarks for various ultrasound (US) transducers. TUT, transparent ultrasound transducer; OUT, opaque ultrasound transducer; and RUT, ring-shaped ultrasound transducer.

	Transducer	f (MHz)	$^{*}\Delta f$ (MHz)	$^{**}\eta(f)$ (dB)	Area (mm^2)	***NEP (μPaHz$^{-1/2}$)	NEP/Δf (μPaHz$^{1/2}$)
Commercial	V308-SU (Olympus, NDT)	5	4.0	-10.5	283	23	5.7
	V212-BC-RM (Olympus, NDT)	20	37.4	-19	28	187	5
	V324-SU (Olympus, NDT)	25	7.6	-13.4	28	99	13
	V214-BC-RM (Olympus, NDT)	50	39.8	-19	28	187	4.7
	V214-BC-RM+Beam combiner	50	39.6	-20	28	210	5.3
Custom-made	Conventional TUT	30	6.4	-9	31	58	9
	Conventional OUT	30	19.3	-9	31	58	3
	RUT	20	9	-11	482	18	2
	Proposed TUT	30	19.3	-9	31	58	3

* Bandwidth
** Efficiency at center frequency
*** Noise-equivalent-pressure

Supplementary Table 5. Simulation parameters used to compare the point spread functions of the custom-made RUT and the proposed TUT.

	Custom-made RUT	Proposed TUT
**Center frequency**	20 MHz	33 MHz
**Aperture**	Outer diameter: 12.7 mm<br>Inner diameter: 5.6mm	5.6 mm x 5.6 mm
**Focal length**	15 mm	
**Speed of sound**	1480 m/s	
**Spatial pulse length**	3 acoustic wavelengths	

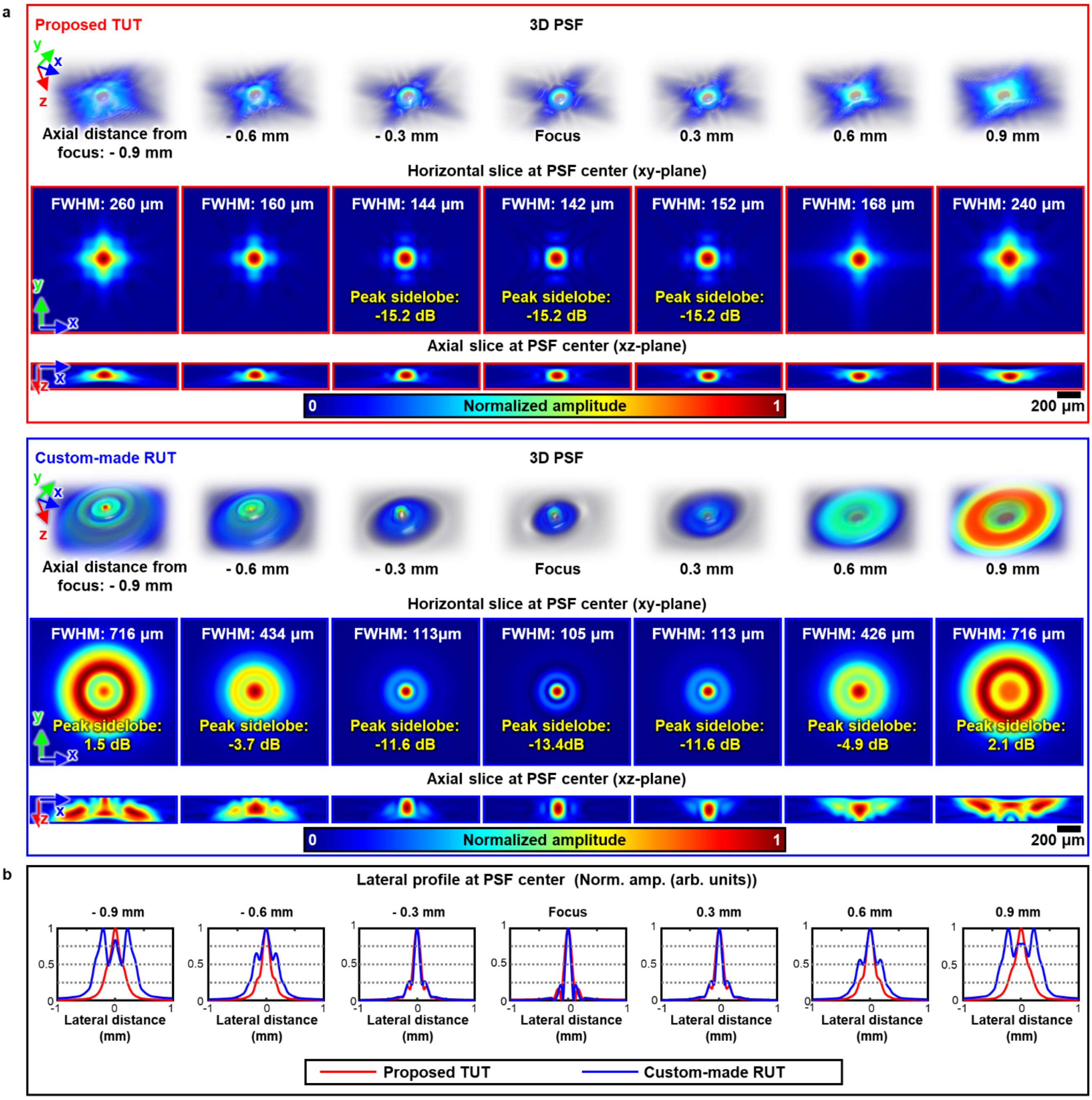

Supplementary Fig. 8. Computational comparison of the acoustic point spread functions (PSFs) of the proposed transparent ultrasound transducer (TUT) and the ring-shaped ultrasound transducer (RUT). a, Simulated PSFs of seven positions, axially spaced at 0.3-mm intervals above and below the focus. **b,** Lateral line profiles of simulated PSFs. Norm. amp, normalized amplitude; and FWHM, Full-width-half-maximum.

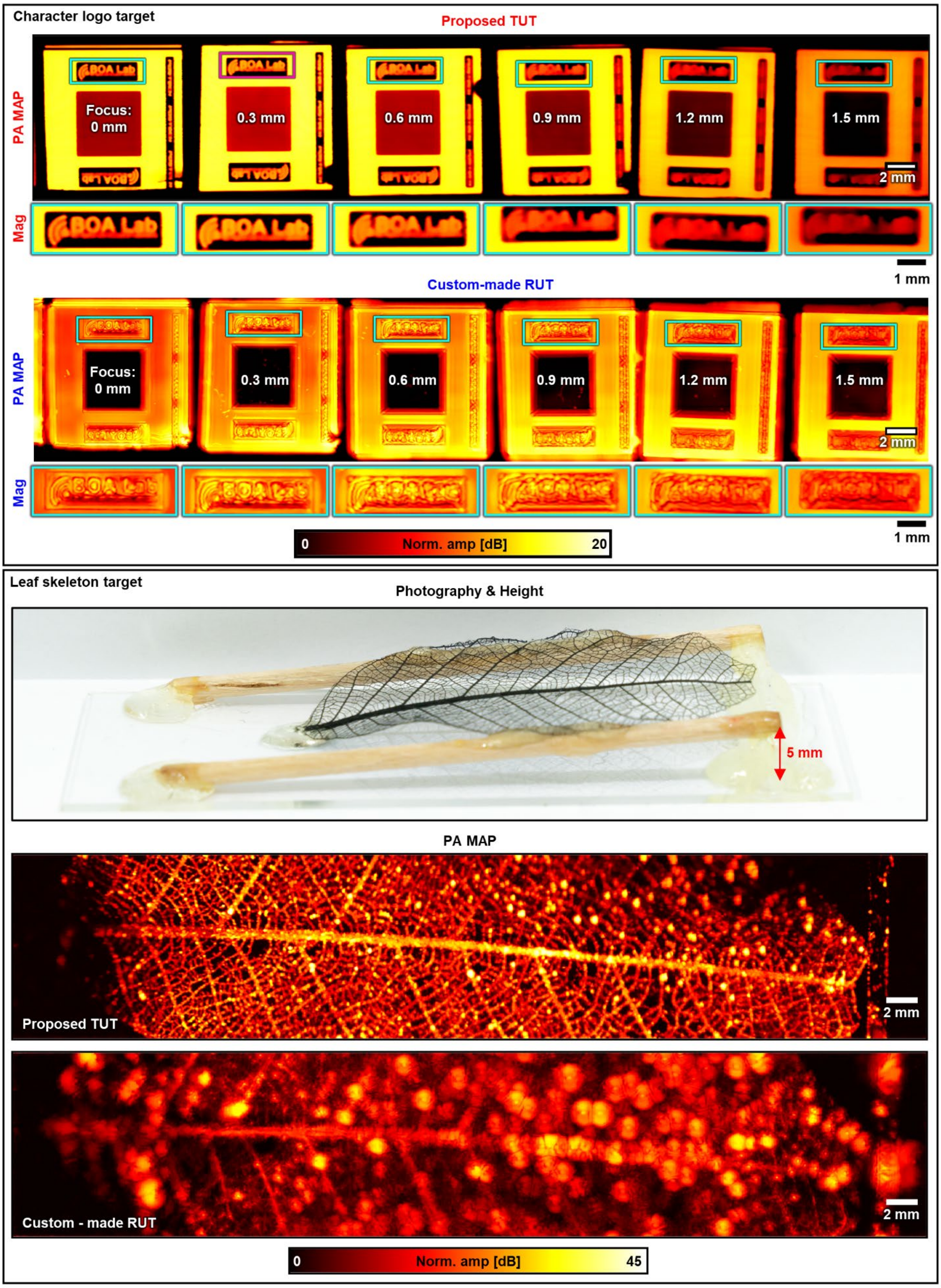

Supplementary Fig. 9. Photoacoustic (PA) imaging of a character logo target phantom and a leaf skeleton target using the proposed transparent ultrasound transducer (TUT) and the ring-shaped ultrasound transducer (RUT). Mag, magnified; and Norm. amp, normalized amplitude.

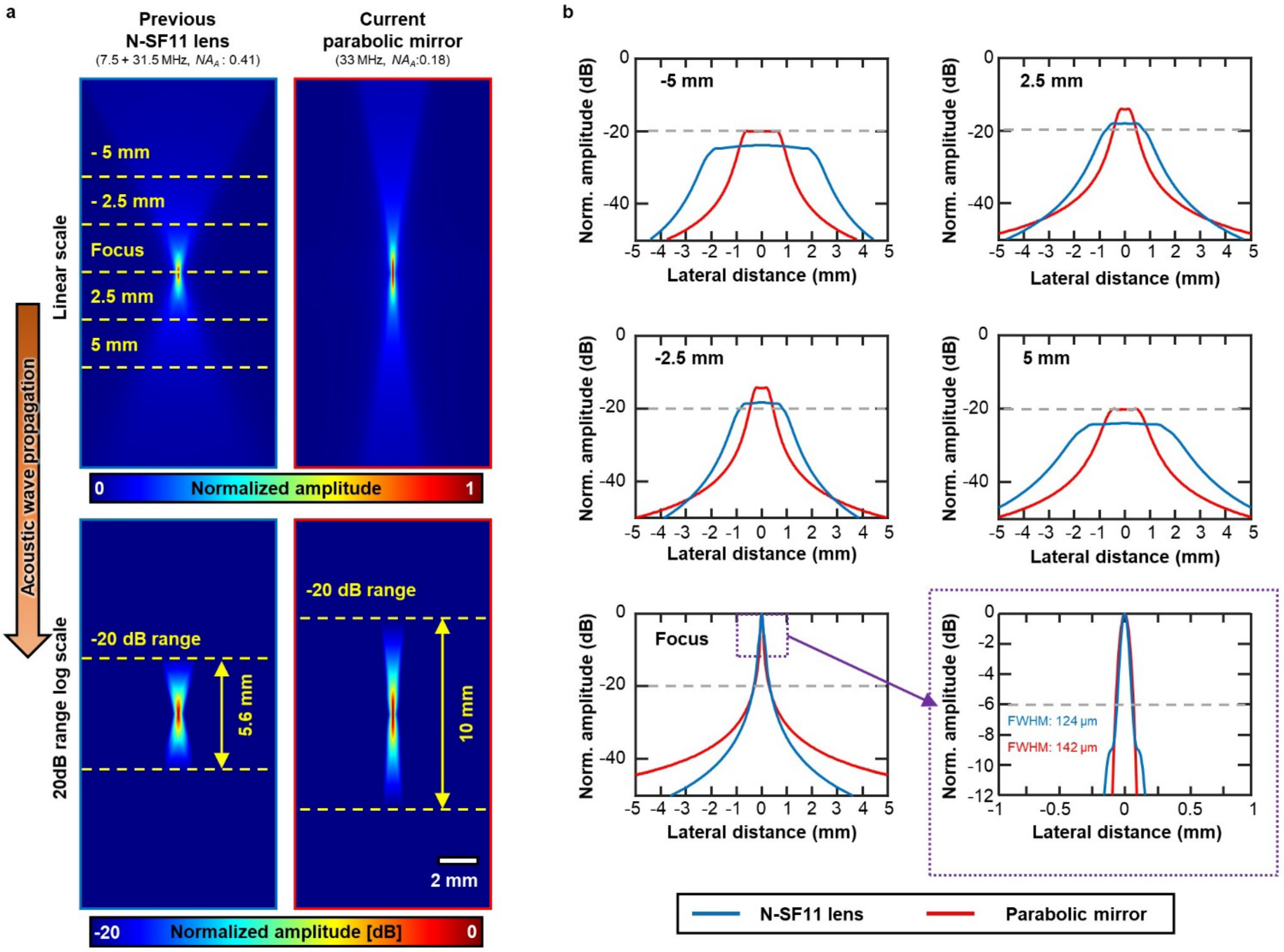

Supplementary Fig. 10. Computational pulsed acoustic field comparison for an N-SF11 concave lens-focused transparent ultrasound transducer (TUT) and the proposed TUT with parabolic mirror focusing. a, Pulse acoustic pressure field projections for the N-SF11 concave lens-focused TUT and the parabolic mirror-focused TUT. **b**, Lateral line profile for selected positions in the pressure field projection. Norm., normalized, and NA_A, acoustic numerical aperture.

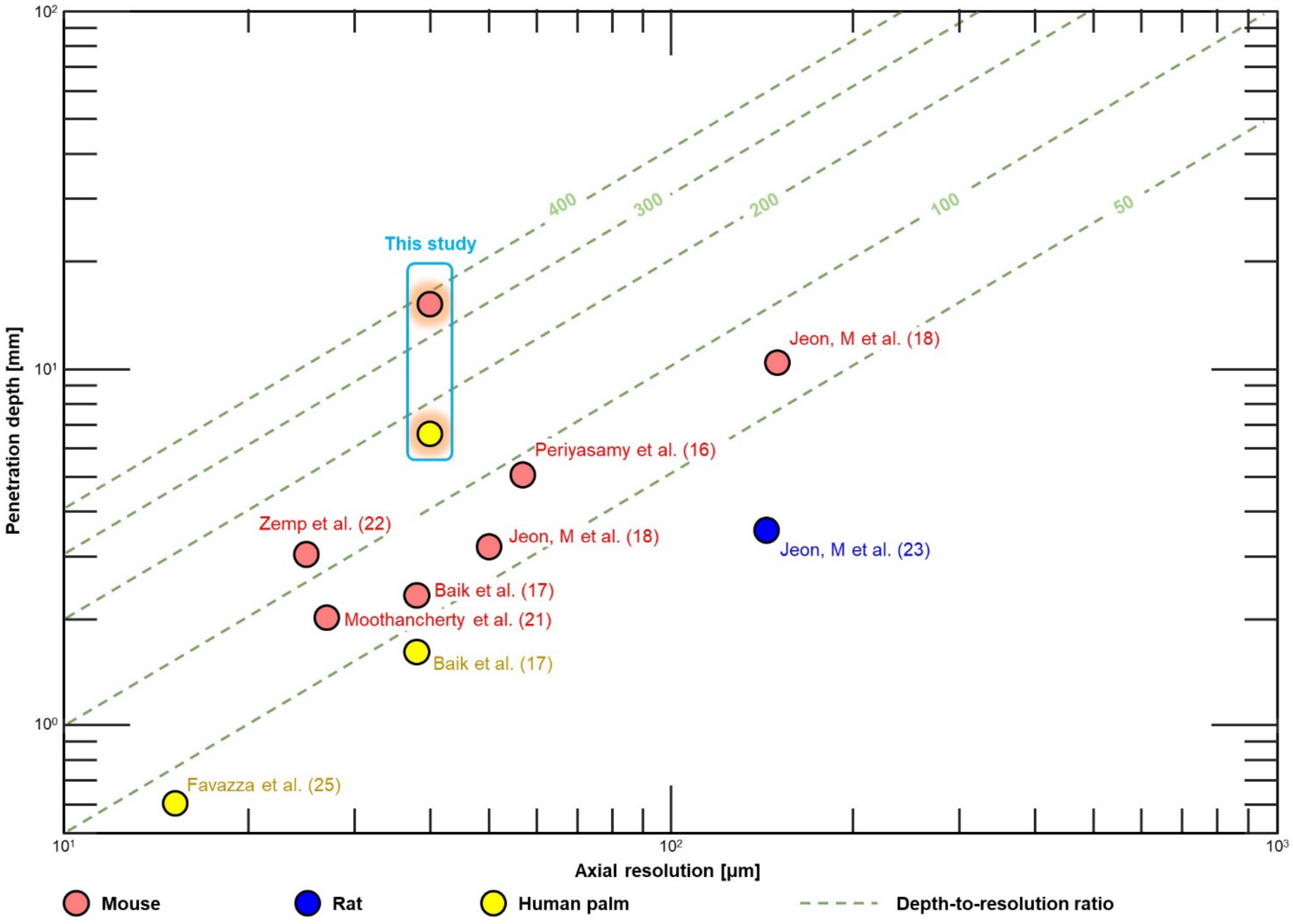

Supplementary Fig. 11. Axial resolutions vs penetration depths of *in-vivo* photoacoustic microscopy studies.

Supplementary Table 6. Summary of performances of acoustic resolution photoacoustic microscopy of chicken breast tissue, a mouse, a rat and a human palm.

	Imaging target [d]	Laser wavelength	Center frequency	Axial resolution	Lateral resolution	Penetration depth	Depth-to-resolution ratio	SNR or CNR	Ultrasound imaging
**This study**	Chicken breast	**1064 nm**	**30 MHz**	**40 µm**	**148 µm**	**13.6 mm**	**340**	**49 dB**	**O**
Moothanchery et al.[12]		532 nm	50 MHz / 75 MHz	27 µm / 18 µm	84 µm / 53 µm	2.7 mm/ 1.8 mm [a]	100	-	X
Sharma et al.[13]		1064 nm	5 MHz	-	-	35 mm	-	-	X
Song et al.[14]		804 nm	5 MHz	144 µm	560 µm	38 mm	263	37 dB	X
Zhang et al.[15]		584, 764 nm	50 MHz	15 µm	45 µm	3.3 mm	220	51 dB[c]	X
Periyasamy et al.[16]		1064 nm	30 MHz	57 µm	130 µm	11 mm	193	58 dB	X
**This study**	Mouse *in-vivo*	**1064 nm**	**30 MHz**	**40 µm**	**148 µm**	**15 mm**	**371**	**49 dB**	**O**
Baik et al.[17]		532 nm	50 MHz	38 µm	85 µm	2.3 mm	61	32 dB	X
Jeon, M et al.[18]		532, 700, 850, 1064 nm	5 MHz / 40 MHz	150 µm / 50 µm	590 µm / 85 µm	10.3 mm/ 3.1 mm	69/62	-	X
Jeon, S et al.[19]		532 nm	50 MHz	-	87 µm	2 mm	-	40 dB	X
Meng et al.[20]		780 nm	25 MHz	-	400–700 µm	3mm	-	-	X
Moothanchery et al.[21]		532 nm	50 MHz	27 µm	84 µm	2 mm	74	-	X
Periyasamy et al.[16]		1064 nm	30 MHz	57 µm	130 µm	5.5 mm[b]	96	58 dB	X
Zemp et al.[22]		578 nm	30 MHz	25 µm	100 µm	3mm[a]	120	-	X
Jeon, M et al.[23]	Rat *in-vivo*	700 nm	5 MHz	144 µm	590 µm	3.5mm[b]	24	-	X
Maslov et al.[24]	Dead rat	532 nm	50 MHz	15 µm	45–120 *µ*m	3 mm	200	50 dB	X
**This study**	Human palm	**1064 nm**	**30 MHz**	**40 µm**	**148 µm**	**6.5 mm**	**163**	**49 dB**	**O**
Baik et al.[17]		532 nm	50 MHz	38 µm	85 µm	1.6 mm	42	32 dB	X
Favazza et al.[25]		584 nm	50 MHz	15 µm	45 µm	0.6 mm	40	-	X

[a], Estimated penetration depth from figure images
[b], Contrast-agent is injected for in vivo imaging.
[c], Contrast to noise ratio
[d], Target whose penetration depth was measured.

Supplementary Note 7. Importance of the acoustic numerical aperture in determining sensitivity and imaging depth

One of the more critical factors in determining the imaging capabilities of USI and PAI is the acoustic numerical aperture (NA_A). An appropriate value should be chosen based on the specific application because high and low NA_A offer different advantages[26]. High NA_A offers excellent lateral resolution and sensitivity, but at the expense of imaging depth. On the other hand, low NA_A provides greater depth for long-range imaging, but at the cost of reduced sensitivity and lateral resolution.

For deep imaging, US typically utilizes a small acoustic NA_A, less than 0.24[2,27,28]. Sensitivity is not a major concern in this scenario because modern US transducers have sufficient sensitivity for imaging with low NA_A. Unlike conventional USI, PAI typically suffers from low detection efficiency due to non-coaxial optical and acoustic beam paths or limited acoustic performance caused by acoustic attenuation and impedance mismatch[11]. For this reason, because the noise-equivalent-pressure in PAI depends on the NA_A[29,30], a high NA_A ($\geq$ 0.44) is commonly used for PA microscopy (PAM) studies[11,15,24,31,32]. Notwithstanding, a high NA_A is not used in USI because it dramatically limits the imaging depth. To extend the imaging depth of PA imaging, it is essential to use high-performance transducers with sufficient sensitivity, even with a low NA_A.

Thanks to the use of transparent matching and backing materials with proper acoustic impedance, the detection efficiency of our TUT is now similar to that of modern conventional OUTs, enabling us to achieve sufficient sensitivity even under low NA_A conditions. Despite our imaging system's NA_A of 0.18, which is 2.5 times worse than in high NA_A ($\geq$ 0.44) imaging systems, our system can still achieve deep penetration with a high signal-to-noise ratio in PA imaging.

Supplementary references

1 Desilets, C. S., Fraser, J. D. & Kino, G. S. The design of efficient broad-band piezoelectric transducers. *IEEE Transactions on sonics and ultrasonics* **25**, 115-125 (1978).
2 Cannata, J. M., Ritter, T. A., Chen, W.-H., Silverman, R. H. & Shung, K. K. Design of efficient, broadband single-element (20-80 MHz) ultrasonic transducers for medical imaging applications. *IEEE transactions on ultrasonics, ferroelectrics, and frequency control* **50**, 1548-1557 (2003).
3 Grewe, M. G., Gururaja, T. R., Shrout, T. R. & Newnham, R. E. Acoustic Properties of Particle Polymer Composites for Ultrasonic Transducer Backing Applications. *Ieee T Ultrason Ferr* **37**, 506-514 (1990). https://doi.org:Doi 10.1109/58.63106
4 Wang, Y. J., Challis, R. E., Phang, A. P. Y. & Unwin, M. E. Bulk Shear Wave Propagation in an Epoxy: Attenuation and Phase Velocity over Five Decades of Frequency. *Ieee T Ultrason Ferr* **56**, 2504-2513 (2009). https://doi.org:10.1109/Tuffc.2009.1337
5 Chen, H. Y. *et al.* Optical-Resolution Photoacoustic Microscopy Using Transparent Ultrasound Transducer. *Sensors-Basel* **19** (2019). https://doi.org:ARTN 5470 10.3390/s19245470
6 Dangi, A., Agrawal, S. & Kothapalli, S. R. Lithium niobate-based transparent ultrasound transducers for photoacoustic imaging. *Opt Lett* **44**, 5326-5329 (2019). https://doi.org:10.1364/Ol.44.005326
7 Chen, R. M. *et al.* Transparent High-Frequency Ultrasonic Transducer for Photoacoustic Microscopy Application. *Ieee T Ultrason Ferr* **67**, 1848-1853 (2020). https://doi.org:10.1109/Tuffc.2020.2985369
8 Park, S., Kang, S. & Chang, J. H. Optically Transparent Focused Transducers for Combined Photoacoustic and Ultrasound Microscopy. *J Med Biol Eng* **40**, 707-718 (2020). https://doi.org:10.1007/s40846-020-00536-5
9 Osman, M. S. *et al.* A Novel Matching Layer Design for Improving the Performance of Transparent Ultrasound Transducers. *IEEE Transactions on Ultrasonics, Ferroelectrics, and Frequency Control* **69**, 2672-2680 (2022).
10 Chen, H. *et al.* A High Sensitivity Transparent Ultrasound Transducer Based on PMN-PT for Ultrasound and Photoacoustic Imaging. *IEEE Sensors Letters* **5**, 1-4 (2021).
11 Park, J. *et al.* Quadruple ultrasound, photoacoustic, optical coherence, and fluorescence fusion imaging with a transparent ultrasound transducer. *P Natl Acad Sci USA* **118** (2021). https://doi.org:ARTN e192087911810.1073/pnas.1920879118
12 Moothanchery, M., Dev, K., Balasundaram, G., Bi, R. & Olivo, M. Acoustic resolution photoacoustic microscopy based on microelectromechanical systems scanner. *Journal of Biophotonics* **13**, e201960127 (2020).
13 Sharma, A., Periyasamy, V. & Pramanik, M. Photoacoustic imaging depth comparison at 532-, 800-, and 1064-nm wavelengths: Monte Carlo simulation and experimental validation. *Journal of Biomedical Optics* **24**, 121904-121904 (2019).
14 Song, K. H. & Wang, L. V. Deep reflection-mode photoacoustic imaging of biological tissue. *Journal of biomedical optics* **12**, 060503-060503-060503 (2007).
15 Zhang, H. F., Maslov, K., Stoica, G. & Wang, L. V. Functional photoacoustic microscopy for high-resolution and noninvasive in vivo imaging. *Nature biotechnology* **24**, 848-851 (2006).
16 Periyasamy, V., Das, N., Sharma, A. & Pramanik, M. 1064 nm acoustic resolution photoacoustic microscopy. *Journal of biophotonics* **12**, e201800357 (2019).
17 Baik, J. W. *et al.* Super wide-field photoacoustic microscopy of animals and humans in vivo. *IEEE transactions on medical imaging* **39**, 975-984 (2019).
18 Jeon, M., Kim, J. & Kim, C. Multiplane spectroscopic whole-body photoacoustic imaging of small animals in vivo. *Medical & biological engineering & computing* **54**, 283-294 (2016).
19 Jeon, S., Park, J., Managuli, R. & Kim, C. A novel 2-D synthetic aperture focusing technique for acoustic-resolution photoacoustic microscopy. *IEEE transactions on medical imaging* **38**, 250-260 (2018).
20 Meng, J. *et al.* Depth-extended acoustic-resolution photoacoustic microscopy based on a two-stage deep learning network. *Biomedical Optics Express* **13**, 4386-4397 (2022).
21 Moothanchery, M. *et al.* High-speed simultaneous multiscale photoacoustic microscopy. *Journal of biomedical optics* **24**, 086001-086001 (2019).
22 Zemp, R., Song, L., Bitton, R., Shung, K. & Wang, L. Realtime photoacoustic microscopy of murine cardiovascular dynamics. *Optics express* **16**, 18551-18556 (2008).
23 Jeon, M. *et al.* Nonionizing photoacoustic cystography with near-infrared absorbing gold nanostructures as optical-opaque tracers. *Nanomedicine* **9**, 1377-1388 (2014).
24 Maslov, K., Stoica, G. & Wang, L. V. In vivo dark-field reflection-mode photoacoustic microscopy. *Opt Lett* **30**, 625-627 (2005).
25 Favazza, C. P., Jassim, O., Cornelius, L. A. & Wang, L. V. In vivo photoacoustic microscopy of human cutaneous microvasculature and a nevus. *Journal of biomedical optics* **16**, 016015-016015-016016 (2011).
26 Lu, J.-y., Zou, H. & Greenleaf, J. F. Biomedical ultrasound beam forming. *Ultrasound in medicine & biology* **20**, 403-428 (1994).
27 Cannata, J. *et al.* in *1999 IEEE Ultrasonics Symposium. Proceedings. International Symposium (Cat. No. 99CH37027).* 1099-1103 (IEEE).

28 Foster, F. S. *et al.* Principles and applications of ultrasound backscatter microscopy. *IEEE transactions on ultrasonics, ferroelectrics, and frequency control* **40**, 608-617 (1993).
29 Winkler, A. M., Maslov, K. & Wang, L. V. Noise-equivalent sensitivity of photoacoustics. *Journal of biomedical optics* **18**, 097003-097003 (2013).
30 Yao, J. & Wang, L. V. Sensitivity of photoacoustic microscopy. *Photoacoustics* **2**, 87-101 (2014).
31 Hu, S., Maslov, K. & Wang, L. V. Second-generation optical-resolution photoacoustic microscopy with improved sensitivity and speed. *Opt Lett* **36**, 1134-1136 (2011).
32 Zhang, H. F., Maslov, K. & Wang, L. V. In vivo imaging of subcutaneous structures using functional photoacoustic microscopy. *Nature protocols* **2**, 797-804 (2007).
