# Supplementary material for: An ultrasensitive and broadband transparent ultrasound transducer for ultrasound and photoacoustic imaging in-vivo
Source: Nat Commun. 2024 Feb 16;15:1444. doi: 10.1038/s41467-024-45273-4 (PMC10873420; doi:10.1038/s41467-024-45273-4)
Supplement: Supplementary file 3 — Description of Additional Supplementary Files [file 41467_2024_45273_MOESM3_ESM.pdf]

Description of Additional Supplementary Information

File name: Supplementary Movie 1

Description: Volumetric ultrasound depth sectioning movie of a live mouse.

File name: Supplementary Movie 2

Description: Volumetric photoacoustic depth sectioning movie of a live mouse.

File name: Supplementary Movie 3

Description: Volumetric ultrasound and photoacoustic overlaid depth sectioning movie of a live mouse.

File name: Supplementary Movie 4

Description: Volumetric ultrasound and photoacoustic depth sectioning movie of a human palm.

File name: Supplementary Code

Description: Original codes for a MATLAB-based KLM model simulator and MATLAB script files for generating simulation data in this study.
